# Start-Up of the Colorectal Cancer Screening Demonstration Program

**Published:** 2008-03-15

**Authors:** Amy DeGroff, Debra Holden, Sonya Goode Green, Jennifer Boehm, Laura Seeff, Florence Tangka

**Affiliations:** Division of Cancer Prevention and Control, National Center for Chronic Disease Prevention and Health Promotion, Centers for Disease Control and Prevention; Research Triangle Institute, Research Triangle Park, North Carolina; Research Triangle Institute, Research Triangle Park, North Carolina; Division of Cancer Prevention and Control, National Center for Chronic Disease Prevention and Health Promotion, Centers for Disease Control and Prevention, Atlanta, Georgia; Division of Cancer Prevention and Control, National Center for Chronic Disease Prevention and Health Promotion, Centers for Disease Control and Prevention, Atlanta, Georgia; Division of Cancer Prevention and Control, National Center for Chronic Disease Prevention and Health Promotion, Centers for Disease Control and Prevention, Atlanta, Georgia

## Abstract

**Introduction:**

In 2005, the Centers for Disease Control and Prevention funded five sites to implement the Colorectal Cancer Screening Demonstration Program (CRCSDP). An evaluation is being conducted that includes a multiple case study. Case study results for the start-up period, the time between initial funding and screening initiation, provide details about the program models and start-up process and reveal important lessons learned.

**Methods:**

The multiple case study includes all five CRCSDP sites, each representing a unique case. Data were collected from August 2005 through September 2006 from documents, observations, and more than 70 interviews with program staff and stakeholders.

**Results:**

Sites differed by geographic service area, screening modality selected, and service delivery structure. Program models were influenced by two factors: preexisting infrastructure and the need to adapt programs to fit local service delivery structures. Several sites modeled program components after their National Breast and Cervical Cancer Early Detection Program. Medical advisory boards convened by all sites provided clinical support for developing program policies and quality assurance plans. Partnerships with comprehensive cancer control programs facilitated access to financial and in-kind resources.

**Conclusion:**

The program models developed by the CRCSDP sites offer a range of prototypes. Case study results suggest benefits in employing a multidisciplinary staff team, assembling a medical advisory board, collaborating with local partners, using preexisting resources, designing programs that are easily incorporated into existing service delivery systems, and planning for adequate start-up time.

## Introduction

Colorectal cancer is the second leading cause of cancer-related death in the United States (1). Although strong scientific evidence suggests that regular colorectal cancer screening is effective in helping to reduce incidence and mortality from this disease (2), less is known about how to effectively implement colorectal cancer screening in a population-based setting. In this context, the Centers for Disease Control and Prevention (CDC) funded five sites in August 2005 to implement the Colorectal Cancer Screening Demonstration Program (CRCSDP) for a 3-year period and planned an evaluation to assess its feasibility. The five grantee organizations are the Maryland Department of Health and Mental Hygiene, the Missouri Department of Health and Senior Services, the Nebraska Department of Health and Human Services, Stony Brook University Medical Center, and Public Health – Seattle & King County.

Before funding the CRCSDP, CDC used *Framework for Program Evaluation in Public Health* (3) to develop an evaluation plan with three purposes: 1) understanding program implementation (processes); 2) measuring program effects (outcomes) at the individual client level, and 3) assessing program efficiencies (costs). CDC adopted a goal-based (4), utilization-focused (5) evaluation approach and developed evaluation questions, consistent with the purposes above, for each of eight CRCSDP program goals, which were defined on the basis of the program components. CDC selected three methods to evaluate the CRCSDP: 1) a multiple case study, 2) the collection and analysis of clients' screening and diagnostic services data, and 3) a costs and cost-effectiveness analysis. CDC is collecting and analyzing data for two distinct periods: 1) program start-up (i.e., the time between initial funding and the initiation of screening services) and 2) screening implementation. This report summarizes case study results for the start-up period, describes the five unique program models and the start-up process, and identifies important lessons learned.

## Methods

The study team conducted a multiple case study to better understand program implementation processes and to describe the experience and context of each CRCSDP program. A multiple case study approach was used in part because it would allow comparisons between the five sites. All five CRCSDP programs were included in the multiple case study (6,7), each representing a unique case. Table 1 presents the eight CRCSDP program goals and offers examples of evaluation questions addressed by the case study.

### Data collection

The study team collected data from documents, interviews, and observations from August 2005 through September 2006. Key documents were summarized by using a structured guide, and other documents were retained in their entirety. Documents included funding proposals to CDC for the first 2 years of the CRCSDP program, program policies, patient flowcharts, and minutes from an all-site conference call. In February and March 2006, the team conducted a telephone interview, using a semistructured interview guide, with the program director for each site; three in-person interviews were also conducted with CDC program consultants who provided technical assistance and other support to the sites.

The team made 2-day visits to each program site during summer 2006 to record observations and conduct interviews with staff and stakeholders. Ten unique, semistructured interview guides were developed for the following positions: bureau chief, program director, program coordinator, quality assurance coordinator, outreach coordinator, epidemiologist, medical advisory board (MAB) member, provider site coordinator, endoscopist, and Comprehensive Cancer Control (CCC) coordinator or other partner. The team identified these roles on the basis of typical staffing patterns among the sites and program policies imposed by CDC (e.g., programs must convene an MAB). Interview questions were developed on the basis of the role of the interviewee, the evaluation questions, and information gathered during the earlier interviews with program directors and CDC program consultants. The team used purposeful sampling to select interviewees who were likely to provide the most in-depth information (5); relevant stakeholders were identified with assistance from program staff. A team of two evaluators conducted most interviews, which were audiotaped and lasted approximately 60 minutes. The team conducted a total of 67 interviews (30 staff and 37 stakeholders). On the basis of informal observations conducted at all sites, descriptive field notes were developed.

### Analysis

Data analysis involved an iterative approach whereby team members regularly met to discuss impressions, review field notes, identify themes, and consider areas of emphasis for subsequent interviews (8). The team transcribed all interviews and entered them along with documents and document summaries into Atlas.ti (Atlas.ti Scientific Software Development GmbH, Berlin, Germany), a software program for qualitative data analysis. Categories and themes were developed both inductively from the data (e.g., challenges in recruiting endoscopists) and deductively from the evaluation questions (e.g., description of partnership activities).

The team developed and refined a codebook with detailed code definitions. A single evaluator was assigned to code all interviews for one program site. The team coded 65 of the 67 on-site interviews, excluding two interviews because the interviewees were unfamiliar with details of their sites' CRCSDP program. Because of resource limitations, the documents were not coded, nor were the five telephone interviews with program directors or the three interviews with program consultants, but these materials were used in the analysis.

The team met twice weekly during the coding process to discuss issues and review the memos of each team member. A second team member coded half of all interviews for each site; the two coders discussed discrepancies to make final coding decisions. The constant comparative method (9) was used to compare categories of data at different levels. Inferences from the coded data were made using content analysis (10). The team developed typologies (e.g., classifying service delivery models) and tables as an additional way to understand the organizational arrangements and service delivery processes (11). Finally, within-case analysis (6) was conducted for each of the five programs, and case-specific reports were developed.

### Credibility

Each member of the evaluation team engaged in all aspects of data collection and analysis, an approach that contributed to a thorough and holistic understanding of each case. Both methodologic and data-source triangulation were used to verify findings; using more than one source of evidence is known to strengthen findings (11-14). The team maintained a detailed audit trail documenting the research methods and process to ensure transparency (13). Finally, the process of member checking was used for the in-case analysis (12,15); this process engages research participants in a review of tentative findings to verify their accuracy.

## Results

We present results for two distinct areas. The first, program models, summarizes characteristics of each CRCSDP program model. The second, program processes, presents data related to key start-up processes.

### Program models

The five sites differed in geographic service area: two served a city (Baltimore, Maryland, and St. Louis, Missouri); two served counties (Suffolk County, New York; and King, Clallam, and Jefferson counties, Washington); and one served a state (Nebraska).

Missouri, Nebraska, and Washington planned to use the guaiac-based fecal occult blood test (FOBT) as the primary screening test, with colonoscopy being used for diagnosis and screening of high-risk people (Table 2). Maryland and New York planned to use colonoscopy as their primary screening test. On the basis of CDC guidelines related to the priority population for the program, we found consistency between the populations served by the five programs.

**Figure 1:**
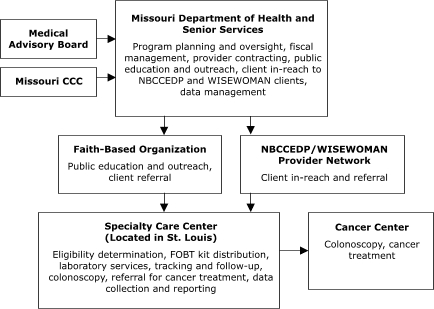
Centralized provider system for the Colorectal Cancer Screening Demonstration Program, Missouri. Both the specialty care center and cancer center provide endoscopic services. CCC indicates Comprehensive Cancer Control; NBCCEDP, National Breast and Cervical Cancer Early Detection Program; WISEWOMAN, Well-Integrated Screening and Evaluation for Women Across the Nation; FOBT, fecal occult blood test.

**Figure 2:**
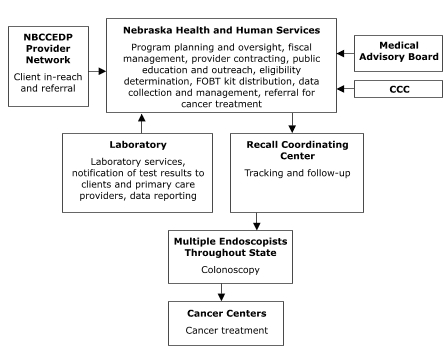
Centralized provider system for the Colorectal Cancer Screening Demonstration Program, Nebraska. NBCCEDP indicates National Breast and Cervical Cancer Early Detection Program; FOBT, fecal occult blood test; CCC, Comprehensive Cancer Control.

**Figure 3:**
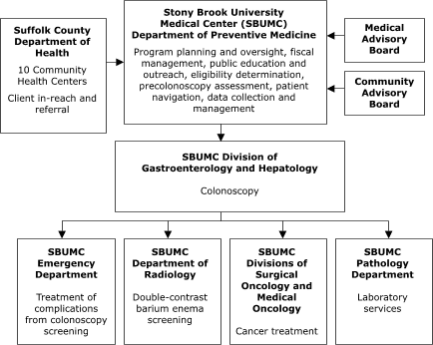
Centralized Provider System for the Colorectal Screening Demonstration Program, New York.

**Figure 4:**
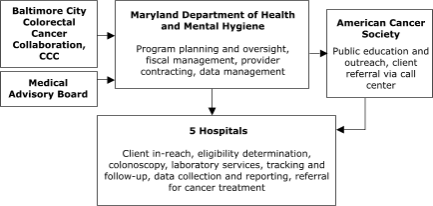
Decentralized provider system for the Colorectal Cancer Screening Demonstration Program, Maryland. CCC indicates Comprehensive Cancer Control.

**Figure 5:**
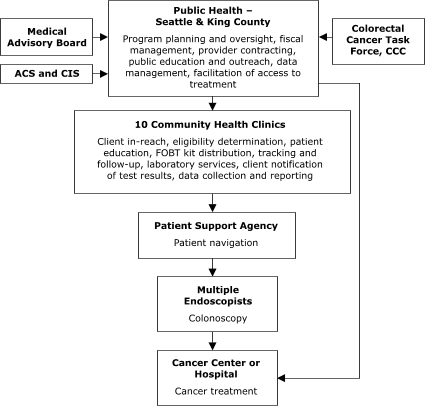
Decentralized provider system for the Colorectal Cancer Screening Demonstration Program, Washington. ACS indicates American Cancer Society; CIS, Cancer Information System; CCC, Comprehensive Cancer Control; FOBT, fecal occult blood test. The Colorectal Cancer Task Force is a subcommittee of the statewide CCC that was established to address colorectal cancer issues.

The organizational relationships for the programs' service delivery systems varied (Figures 1–5). Nebraska and New York planned to deliver screening services themselves. Maryland, Missouri, and Washington, however, planned to provide program oversight and contract with other agencies to deliver screening services. Missouri, Nebraska, and New York planned centralized service delivery systems, but Maryland and Washington planned decentralized systems.

The Missouri Department of Health and Senior Services planned to contract with a provider in St. Louis to assess client eligibility for screening, deliver FOBT services, track and follow up on clients, and provide colonoscopies (Figure 1). The Nebraska Department of Health and Human Services planned to assess client eligibility for screening and deliver FOBT services, but to contract with outside providers for tracking and follow-up, laboratory, and colonoscopy services (Figure 2). Stony Brook University Medical Center in New York represents an enclosed system in which departments within the medical center planned to conduct all aspects of service delivery (Figure 3).

Of the sites with decentralized models of service delivery, the Maryland Department of Health and Mental Hygiene (Figure 4) planned to contract with five hospitals, each of which would provide all elements of screening service. In Washington, Public Health – Seattle & King County planned to contract with 10 primary care centers to assess screening eligibility, deliver FOBT services, ensure tracking and follow-up, and provide laboratory services (Figure 5). The plan also called for contracting with 1) another agency to provide patient navigation services to people referred for colonoscopy and 2) several endoscopists to conduct colonoscopy. In general, staff members in Maryland and Washington valued the decentralized model for its community-based orientation but perceived the model as more difficult to establish because of the need to support multiple sites in integrating and adapting the program into their existing service delivery systems.

Two key factors influenced the program design of all five programs. First, several sites developed the new CRCSDP, or components of it, from existing programs such as the National Breast and Cervical Cancer Early Detection Program (NBCCEDP). For instance, sites planned to use NBCCEDP provider networks to support client in-reach or to distribute FOBT kits and were in the process of integrating other program components with existing NBCCEDP components. One staff member noted, "The easy part for us was having a screening and tracking system in place already that we were comfortable with [NBCCEDP]. . . . We were able to use similarities in our existing system and customize those for CRCSDP." The second factor influencing the CRCSDP program models was the need for sites to tailor service delivery systems in ways that facilitated their integration into existing clinical structures. Participants said such integration was necessary to minimize the burden and disruption for participating clinical sites. For decentralized models, the need to "fit" the provider context resulted in unique patient flow patterns at multiple provider settings.

### Start-up processes

The start-up process lasted 9 to 11 months and involved assembling a staff team, developing program models, convening a MAB to assist in developing policies and procedures, building partnerships, planning for client recruitment, developing a data management system, and identifying resources for the treatment of complications.

**Staffing**

Each program recruited a team of two or three people, usually from existing positions, to assist in developing the new program. Teams typically included a program director, a program coordinator working on day-to-day activities, and a data management specialist. Programs that were able to easily access staff with clinical expertise within the grantee organization noted the importance of being able to do so. Nearly all CRCSDP program directors were also managing their state or region's NBCCEDP, and some were managing a Well–Integrated Screening and Evaluation for Women Across the Nation (WISEWOMAN) program, another CDC-funded screening program (16). Program directors had extensive program and management experience and preexisting partner relationships with cancer prevention and control leaders in their state. The team approach helped ensure that enough people with varied expertise were available to attend to the many start-up responsibilities.

**Medical advisory board**

An MAB was convened by each program and provided essential clinical guidance during the start-up period, especially for CRCSDP sites lacking staff with extensive medical expertise in colorectal cancer. MAB composition varied by site but largely reflected clinical disciplines relevant to colorectal cancer and screening, including primary care specialists, gastroenterologists, and radiologists (Table 3). One respondent suggested that the prescription for a well-rounded MAB includes "basically anybody involved in any step of the way from screening to diagnosis to treatment, a continuum of care, with a heavy emphasis on GI [gastrointestinal specialists]." The MABs served as a functional work group, providing direction on policy development, program eligibility criteria, patient flow, data collection, and quality assurance. MABs participated informally, meeting as a group infrequently but otherwise being accessible to program staff by telephone and e-mail.

**Partnerships**

Partnerships provided critical resources, both financial and in-kind, and played an active role during program start-up. Key partners included state or regional CCC groups, the American Cancer Society (ACS), community-based organizations, and universities. Several partners provided in-kind staff support, and CCC groups contributed financial resources for a public education campaign in one site and database development in two others. CCC groups were also valuable in negotiating relationships with MAB members, endoscopists, and representatives of clinical provider sites. An ACS call center planned to recruit CRCSDP clients for one program, and a local university planned to assist with client recruitment and evaluation in another.

**Client recruitment**

CRCSDP sites planned public education, outreach, and in-reach strategies to recruit clients for screening (Table 4). Several sites adopted CDC's Screen for Life: National Colorectal Cancer Action Campaign or ACS public education materials. Staff emphasized the use of culturally sensitive public education materials. Although public education efforts were intended to raise awareness about, and create demand for, the new CRCSDP, interviewees expressed apprehension about creating too great a demand for screening services early in program implementation. Staff planned to begin with a slow process of recruitment so they could test their systems. Ten Suffolk County community health centers collaborated with the New York program during the start-up period to develop a plan for referring clients for screening. Other CRCSDP sites focused on developing in-reach efforts to recruit clients from existing screening programs such as the NBCCEDP. However, interviewees expressed concerns about recruiting men for the CRCSDP through NBCCEDP, observing that men generally are less likely to access preventive health care services. One stakeholder noted the following: "All of the people from the men's health sector say that the only thing men say is that 'my wife made me do it' [get screened]. All of the doctors say that, too, that men say their wives made them come in. But we don't want to put all of that burden on women. Women are used to getting screenings and doing preventive care; it's not part of the culture for men."

**Data management systems**

During program start-up, CDC, in collaboration with the five CRCSDP sites, developed a set of colorectal clinical data elements to collect patient-level demographic, screening, and diagnostic data on program clients. Whereas one CRCSDP site developed a new data system, others augmented existing systems (e.g., the NBCCEDP data system) to integrate the data elements. With support from MABs and provider sites, each program also developed data collection forms (e.g., patient enrollment, health history, FOBT screening). Although staff suggested that the development of data systems and forms was not particularly difficult, they observed that it was an especially time-consuming component of the start-up period.

**Treatment resources**

Staff identified challenges in securing resources for cancer treatment. Because CDC funds cannot be used for treatment (17), programs depended on soliciting in-kind support or charity care from a provider system viewed by staff as already overburdened.

## Discussion

The program models and start-up process of the CRCSDP offer valuable insight to those with an interest in developing colorectal cancer screening programs. Several key factors emerged from the evaluation of the start-up experience of the five sites studied here. These factors include use of a multidisciplinary team, involvement of an MAB, relationships with partners, the use of preexisting resources, a program model that fits existing service delivery systems, and adequate planning time.

In these five programs, two to three staff with expertise in program management and administration (e.g., collaboration, contracting, policy development), program coordination (e.g., day-to-day management, training, support), and data management (e.g., data systems, data form development) provided an adequate team for program start-up. Clinical expertise and comfort discussing clinical issues with MAB members and service providers were important skills for the management team.

Access to clinicians with expertise in colorectal cancer was essential to start-up. A well-rounded MAB that included professionals in disciplines related to the screening process (e.g., endoscopists, pathologists, radiologists, surgical oncologists, social workers, community-based practitioners) was beneficial.

CDC and other organizations recognize that public health problems demand collaborative efforts rather than "going it alone" (18,19). Active and extensive partnerships were fundamental in helping the programs plan to recruit clients, increase public awareness about the need for screening, and facilitate relationships with MAB members and screening sites.

The five CRCSDP sites leveraged existing resources to build a new colorectal cancer screening program. Partner agencies (e.g., CCC, ACS), other screening programs (e.g., NBCCEDP), and internal agency departments (e.g., health communications, epidemiology) helped reduce costs and support program development. The length of time needed to develop data systems and data collection forms suggests new programs may benefit from using existing data forms and data collection sets.

These five programs used program models that would most easily integrate into existing service delivery systems. For the decentralized models, integration involved allowing for varied implementation approaches within multiple service delivery sites for the same program (e.g., five different clinical sites providing colonoscopy screening). Reliance on in-reach to NBCCEDP clients and overall concerns about effectively recruiting men suggest programs may need to consider program models that include unique recruitment efforts for men.

Although CDC had anticipated a 6-month start-up period, these programs needed 9 to 11 months to hire staff, convene an MAB, develop policies, build partnerships, organize a service delivery system, plan for client recruitment, secure treatment resources, and develop data management systems. One staff member advised, "The devils are in the details — all the little things that you have to think through that we didn't even think of — things we thought we knew but we didn't."

The CRCSDP evaluation team will continue to work with the five sites as they provide colorectal cancer screening to low-income, underserved communities. The case study, in particular, contributes to important process evaluation efforts that improve our understanding of the CRCSDP's program operations, implementation, and service delivery (20). Recognizing that the potential for evaluation to effect change is dependent on its use (21), evaluators encourage others with an interest in colorectal cancer screening to consider the results presented here.

## Figures and Tables

**Table 1:** Program Goals and Examples of Evaluation Questions Related to Program Start-Up, Colorectal Cancer Screening Demonstration Program, 2006

Goal	Evaluation Questions Related to Program Start-Up
Provide sound program planning and management.	What staffing is used during program start-up? How are medical advisory boards comprised? What is their role during program start-up?
Develop and maintain effective partnerships to ensure sustainability.	What partnerships have been developed to support the program?
Effectively recruit low-income, medically underserved participants for colorectal screening through public education and outreach.	What priority populations are proposed to be reached? What types of recruitment strategies are planned?
Increase the rate of colorectal cancer screening among low-income, medically underserved populations.	Not applicable in this phase.
Provide program recipients with appropriate screening and rescreening services.	What is the start-up time for programs? How is the provider system structured for services delivery?
Assure program recipients receive appropriate diagnosis and treatment services.	How will patient navigation services be provided? How have programs secured treatment services for clients diagnosed with cancer?
Conduct monitoring, tracking, and evaluation activities.	What types of data systems have been developed by programs?
Provide cost-effective services.	What in-kind contributions have been secured by programs?

**Table 2:** Characteristics of Program Models, Colorectal Cancer Screening Demonstration Program, 2006

CRCSDP Program	Service Area	Test Type	Service Delivery Model	Provider Network
Maryland	Baltimore City	Colonoscopy	Decentralized	5 hospitals
Missouri	St. Louis	Fecal occult blood test (FOBT)	Centralized	1 specialty care center and 1 medical center
Nebraska	Statewide	FOBT	Centralized	State health department
New York	Suffolk County	Colonoscopy	Centralized	1 university medical center
Washington	King, Clallam, and Jefferson counties	FOBT	Decentralized	10 community health centers

**Table 3:** Composition and Start-Up Activities of Medical Advisory Boards, Colorectal Cancer Screening Demonstration Program (CRCSDP), 2006

Disciplines Represented by MABs	MAB Start-Up Activities
Gastroenterologists Pathologists Surgical oncologists Surgeons Other physicians Radiologists Physician assistants Nurse practitioners Social workers Partner representatives (ACS, CCC) Health department representatives Provider site representatives	Reviewed CDC policies for the CRCSDP Developed program-specific policies Determined eligibility criteria Advised on screening test, procedures, and bowel preparation materials Advised on patient flow process and quality assurance Reviewed patient-level data variables and data collection forms developed for the program Developed plans to treat patients experiencing medical complications from screening or diagnostic procedures Provided guidance for professional education Reviewed patient education materials Advocated for provider participation and facilitated relationships with medical institutions Advised on treatment issues and advocated for treatment resources Promoted the program in the colorectal cancer and larger cancer community

MAB indicates medical advisory board; CDC, Centers for Disease Control and Prevention; ACS, American Cancer Society; CCC, Comprehensive Cancer Control.

**Table 4:** Public Education, Outreach, and In-Reach Strategies by Site, Colorectal Cancer Screening Demonstration Program (CRCSDP), 2006

CRCSDP Site	Public Education	Outreach	In-Reach
Maryland	ACS No Excuses campaign	ACS call-in center with referral to provider sites.	Provider site in-reach through other existing screening programs (e.g., prostate, breast, cervical); referral from federally qualified health centers affiliated with provider sites.
Missouri	CDC Screen for Life campaign used via television and radio advertisements	Outreach through a faith-based organization, bus signs, posters in laundromats and grocery stores, peer health worker program.	NBCCEDP providers refer potential clients to provider site; mailings to NBCCEDP clients and their partners.
Nebraska	Public education materials adopted from Screen for Life, ACS, and NIH's Cancer Information Services	Extensive focus group testing conducted to shape messaging; plans for more targeted outreach through events (e.g., farm auctions).	NBCCEDP providers refer potential clients to state health department.
New York	Screen for Life posters, fact sheets, and brochures placed in community health centers	Video developed to use in community health clinic waiting rooms.	10 Suffolk County community health clinics assess initial eligibility and refer to the provider site.
Washington	ACS materials, CRCSDP brochure developed in English, Spanish, and Vietnamese	No activities planned.	Provider site (primary care clinics) in-reach to NBCCEDP clients and other eligible clients; incentive gift cards for clients.

ACS indicates American Cancer Society; CDC, Centers for Disease Control and Prevention; NBCCEDP, National Breast and Cervical Cancer Early Detection Program; NIH, National Institutes of Health.

## References

[1] (2007). U.S. Cancer Statistics Working Group. United States cancer statistics: 2003 incidence and mortality.

[2] Mandel JS, Church TR, Bond JH, Ederer F, Geisser MS, Mongin SJ (2000). The effect of fecal occult-blood screening on the incidence of colorectal cancer. N Engl J Med.

[3] (1999). Framework for program evaluation in public health. MMWR Recomm Rep.

[4] Stufflebeam DL (2001). Evaluation models. New Dir Eval.

[5] Patton MQ (2002). Qualitative research & evaluation methods.

[6] Stake RE (1995). The art of case study research.

[7] Stake RE (2006). Multiple case study analysis.

[8] Bogdan RC, Biklen SK (2007). Qualitative research for education: an introduction to theories and methods.

[9] Glaser BG, Strauss AL (1967). The discovery of grounded theory: strategies for qualitative research.

[10] Krippendorf K (1980). Content analysis: an introduction to its methodology.

[11] Miles MB, Huberman AM (1994). Qualitative data analysis: an expanded sourcebook.

[12] Creswell JW, Miller DL (2000). Determining validity in qualitative inquiry. Theory into Practice.

[13] Lincoln YS, Guba EG (1985). Naturalistic inquiry.

[14] Mathison S (1988). Why triangulate?. Educational Researcher.

[15] Merriam SB (1998). Qualitative research and case study applications in education.

[16] WISEWOMAN — Well–Integrated Screening and Evaluation for Women Across the Nation.

[17] (2005). Colorectal Cancer Screening Demonstration Program. Fed Regist.

[18] CDC health protection goals fact sheet: goals for the 21st century.

[19] (2003). Institute of Medicine. The future of the public's health in the 21st century.

[20] Rossi PH, Freeman HE, Lipsey MW (1999). Evaluation: a systematic approach.

[21] Weiss CH (1998). Evaluation.

